# A Real-Time Atrial Fibrillation Detection Algorithm Based on the Instantaneous State of Heart Rate

**DOI:** 10.1371/journal.pone.0136544

**Published:** 2015-09-16

**Authors:** Xiaolin Zhou, Hongxia Ding, Wanqing Wu, Yuanting Zhang

**Affiliations:** 1 CAS/CUHK Research Centre for Biosensors and Medical Instruments, Shenzhen Institutes of Advanced Technology, Chinese Academy of Sciences, Shenzhen, Guangdong, China; 2 The Key Laboratory for Health Informatics of the Chinese Academy of Sciences, Shenzhen Institutes of Advanced Technology, Chinese Academy of Sciences, Shenzhen, Guangdong, China; 3 Department of Electronic Engineering, The Chinese University of Hong Kong, Shatin, N.T., Hong Kong, China; University of Minnesota, UNITED STATES

## Abstract

Atrial fibrillation (AF), the most frequent cause of cardioembolic stroke, is increasing in prevalence as the population ages, and presents with a broad spectrum of symptoms and severity. The early identification of AF is an essential part for preventing the possibility of blood clotting and stroke. In this work, a real-time algorithm is proposed for accurately screening AF episodes in electrocardiograms. This method adopts heart rate sequence, and it involves the application of symbolic dynamics and Shannon entropy. Using novel recursive algorithms, a low-computational complexity can be obtained. Four publicly-accessible sets of clinical data (Long-Term AF, MIT-BIH AF, MIT-BIH Arrhythmia, and MIT-BIH Normal Sinus Rhythm Databases) were used for assessment. The first database was selected as a training set; the receiver operating characteristic (ROC) curve was performed, and the best performance was achieved at the threshold of 0.639: the sensitivity (*Se*), specificity (*Sp*), positive predictive value (*PPV*) and overall accuracy (*ACC*) were 96.14%, 95.73%, 97.03% and 95.97%, respectively. The other three databases were used for independent testing. Using the obtained decision-making threshold (i.e., 0.639), for the second set, the obtained parameters were 97.37%, 98.44%, 97.89% and 97.99%, respectively; for the third database, these parameters were 97.83%, 87.41%, 47.67% and 88.51%, respectively; the *Sp* was 99.68% for the fourth set. The latest methods were also employed for comparison. Collectively, results presented in this study indicate that the combination of symbolic dynamics and Shannon entropy yields a potent AF detector, and suggest this method could be of practical use in both clinical and out-of-clinical settings.

## Introduction

Cardiovascular disease is the leading cause of death globally, and the atrial fibrillation (AF) is one of the most prevalent cardiac arrhythmias in the elder people [1], [2], [3]. The Framingham Heart Study indicated the lifetime risks for development of AF are one in four for adults over the age of 40 years based on the study population which involved 3999 men and 4726 women who were followed up over a 32-year period from 1968 to 1999 [4]. Because of hemodynamic effects, the AF causes intracardiac-blood stasis which in return may predispose the formation of clots (thrombus). These blood clots may break loose and can be washed into the brain, where they may trigger a fatal stroke. Indeed, AF is associated with a fourfold to fivefold increase in the risk of stroke and the AF-related strokes (i.e., ischemic-type stroke) tend to be severe, with a major impact on morbidity and mortality [1], [5], [6]. Up to three million people worldwide suffer the AF-related strokes each year, and the total number of strokes are increasing because of the aging population [7–9]. Therefore, the early identification of AF is a necessary step for averting the possibility of blood clotting, and ischemic stroke.

To deal with the computerized AF-detection issue several research studies consider processing the electrocardiograms (ECGs) over the past decades since the AF is characterized by poorly coordinated atrial activation (AA) of heart and turbulent cardiac beating [10–20]. Most of these studies based on the RR (R-wave peak to R-wave peak) interval irregularity (RRI) in ECGs. A very few studies were implemented with reference to the replacement of consistent P waves by rapid oscillations or F waves that alter in amplitude, morphology, and timing as a result from the abnormal AA [2]. Although the diversification information of P wave or rapid AA can be an alternative clue in the identification of AF episodes, it is impossible to pinpoint the independent P wave accurately because original ECG signals may be corrupted with various types of high-intensity noise while the P wave is general of very low-intensity magnitude, which may incur many false classification for episodes. In addition, the relationship between the rapid AA and diversification information of P wave in the surface ECG to the diverse mechanisms of AF has not yet been well elucidated [3]. Because of the challenges in detecting AA, the detection methods based on inferences from RRI are preferred to produce relatively more precise identification of AF since the R-wave peak of QRS complex is the most prominent characteristic feature of an ECG recording and the least susceptible to various kinds of noise [14–16, 21]. However, when evaluated on different types of clinical data, the performance of some algorithms in this category is not ideal and each can be improved [22]. Some algorithms are too complicated to be realized in real-time applications and consequently unsuitable for use in wearable devices.

We herein describe a real-time and low-complexity but robust method for the discrimination of AF episodes in surface ECGs. This method first generates a symbolic sequence by defining the heart rate (HR) values into different instantaneous states with a fixed interval. The symbol sequence is subsequently converted into a word sequence using a novel operator. The probability distribution of the word sequence in the specified space is obtained, and a coarser version of Shannon entropy (SE) is next employed to discriminate the AF arrhythmias. An important feature of this method is that it is different from previous approaches because the proposed method is based on the HR which is firstly introduced in the field of AF detection. The performance of this newly proposed method is systematically investigated on four well-characterized and representative clinical databases under various experimental situations. We also calculate sensitivity, specificity, positive predictive value, overall accuracy and compare these parameters with the latest AF detection methods.

## Materials and Methods

The AF detection algorithm consists of three steps: 1) HR sequence is converted to a symbolic sequence in a fixed interval; 2) a probability distribution is constructed from the word sequence which is transformed from the symbolic sequence; 3) a coarser version of Shannon entropy is employed to quantify the information size of HR sequence using the probability distribution of word sequence, and then differentiates the AF episodes.

### Symbolic dynamics of HR sequence

**Fig 1:**
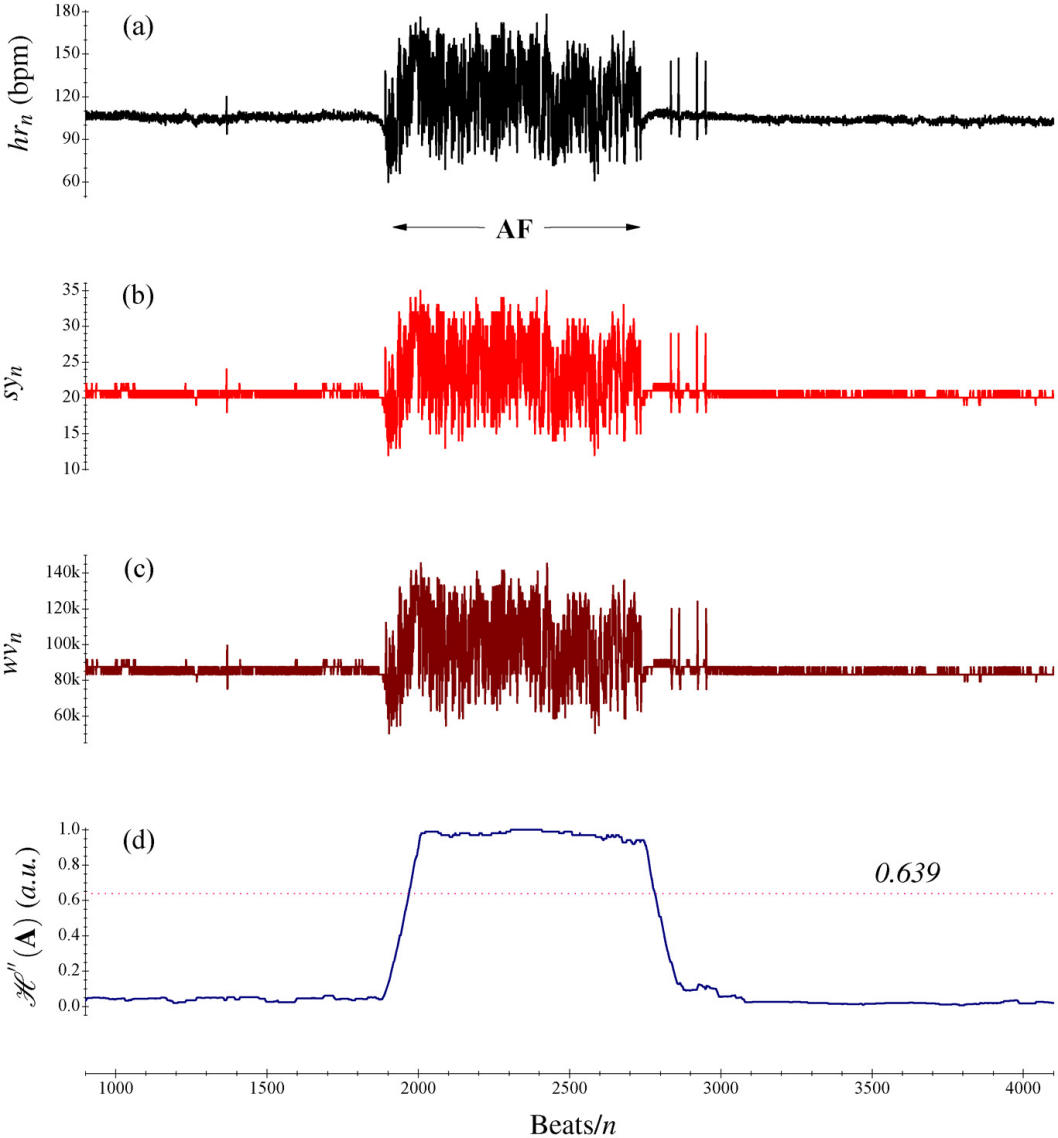
Example for the application of this method for detecting AF. (A) The original HR sequence *hr*_*n*_; (B) The distribution of symbols *sy*_*n*_; (C) The relevant word sequence *wv*_*n*_ of *sy*_*n*_ in (b), and (D) The distribution of SE H" (**A**).

Let *hr*_*n*_ denote the obtained successive HR sequence which can be calculated from RR sequence. The symbolic dynamics is introduced to describe the dynamic behavior of *hr*_*n*_. The symbolic dynamics encodes the information of *hr*_*n*_ to a series with fewer symbols, with each symbol representing an instantaneous state of heart beating. The mapping function of the symbol transform is given by,

$$sy_{n}=\{\begin{array}{ccc} 63 & & \;\;\;\;\text{if}\;\;\;\;hr_{n}⩾315\\ \left\lfloor hr_{n}/5\right\rfloor & & \text{other}\;\text{cases}\;\;\;\;\;\;\;\;\;\;\;\; \end{array}$$ (1)

where ⌊⋅⌋ represents a floor operator. It is apparent that the maximum HR for analysis in the present study is 315 beats per minute (bpm). The raw HR sequence *hr*_*n*_ is thereby transformed into a symbol sequence *sy*_*n*_ with specific symbols from the predefined “alphabet” (i.e., 0 to 63). It should be noted that the maximum value of the total number of instantaneous states in each *hr*_*n*_ sequence is 64. Fig 1(A) and 1(B) depict examples of a *hr*_*n*_ sequence and the corresponding symbols which are transformed by Eq (1).

In order to explore the chaotic behavior of the symbolic series *sy*_*n*_ as explained in the following subsection and to generate more different instantaneous states of HR, we apply the commonly used 3-symbols template (i.e., a word consists of 3 successive symbols) to examine the entropic properties. The word value can then be calculated by a novel operator as defined below,

$$wv_{n}=\left(sy_{n-2}\times 2^{12}\right)+\left(sy_{n-1}\times 2^{6}\right)+sy_{n}$$ (2)

where, *sy*_*n*−2_ × 2^12^ and *sy*_*n*−1_ × 2^6^ are implemented with *sy*_*n*−2_ < < 12 and *sy*_*n*−1_ < < 6, respectively. For instance, the encoded value of 3 successive symbols ‘013’ is 67 = 0 × 4096 + 1 × 64 + 3. By a simple calculation, we can see that the word value *wv*_*n*_ lies within 0 ⩽ *wv*_*n*_ ⩽ 262143 (262143 = 63 × 4096 + 63 × 64 + 63). Fig 1(C) further displays the word sequence of *sy*_*n*_ shown in Fig 1(D). The symbolic dynamics involving Eqs (1) and (2) can also be partially understood as applying a finite impulse response digital filter on *hr*_*n*_ sequence, and the relevant inherent time delay of this filter is 1.5 points with respect to *hr*_*n*_.

### Shannon entropy

Without loss of generality, let **A** = (*A*∣*P*) denote a dynamic system. The characteristic (i.e., unique) elements in **A** can be defined as *A* = {*a*_1_, ⋯, *a*_*k*_} with the corresponding probability set *P* = {*p*_1_, ⋯, *p*_*k*_} (1 ⩽ *k* ⩽ *N*), where *N* and *k* are total number of the elements and characteristic elements in space **A**, respectively. Each element *a*_*i*_ has the probability *p*_*i*_ = *N*_*i*_/*N* (0 < *p*_*i*_ ⩽ 1, $\begin{array}{c} \sum_{i=1}^{k}p_{i}=1 \end{array}$), where *N*_*i*_ is the total number of the specific element *a*_*i*_ in **A**. We thus define a coarser version of Shannon entropy (SE) H" (**A**) to quantitatively calculate the information size of *wv*_*n*_. H" (**A**) is of the form [19],

$$\begin{array}{c} H^{{}^{\prime \prime }}\left(A\right)=-\frac{k}{N\log_{2}N}\sum_{i=1}^{k}p_{i}\log_{2}p_{i} \end{array}$$ (3)

In this study, the dynamic **A** comprises of 127 consecutive word elements from *wv*_*n*−126_ to *wv*_*n*_ (i.e., the bin size in this case is *N* = 127). By determining the characteristic set *A* and the relevant probability set *P* with these elements, we can thus calculate the coarser SE H" (**A**). For each cardiac beat *hr*_*n*_ (and *wv*_*n*_), the AF rhythm is labeled if H" (**A**) meets or exceeds a discrimination threshold, and otherwise the non-AF is decided, which is described in Fig 1(D). In the present study, we utilize a training database to obtain the optimal discrimination threshold by investigating various threshold settings which lie within the range [0.0, 1.0] with an increment of 0.001, the best performing threshold is thus derived and employed from the receiver operating characteristic (ROC) curve for the performance assessment using testing databases.

### Key issues of online processing

Eq (2) can be implemented with a real-time process scheduling algorithm since *sy*_*n*_ and *wv*_*n*_ involve a causal relationship. The other computational challenges lie in the Eq (3) can be overcome with a pre-calculated map of $-\frac{1}{\log_{2}N}p_{i}\log_{2}p_{i}$ and an elaborately designed recursive implementation of Eq (3). That is to say, this AF detector can be realized by recursive methods towards the beat-to-beat, real-time screening.

#### Mapping the definition of $-\frac{1}{\log_{2}N}p_{i}\log_{2}p_{i}$

It is apparent that every characteristic element of each bin *N* may have the probability *p*_*i*_ = *i*/*N* (1 ⩽ *i* ⩽ *N*, i.e., 1/*N* ⩽ *p*_*i*_ ⩽ 1). In light of this observation, a probability array *PiMap* can be pre-defined [19],

$$\begin{array}{cc} PiMap[127] & =-\frac{Cons}{\log_{2}N}\{p_{1}\log_{2}p_{1},\cdots ,p_{63}\log_{2}p_{63},\\ & \;\;\;\;\;\;\;\;\;\;\;\;\;\;\;\;p_{64}\log_{2}p_{64},\cdots ,p_{127}\log_{2}p_{127}\}\\ & \overset{\left\lfloor \cdot \right\rfloor }{=}\left\{7874,\cdots ,71790,71291,\cdots ,0\right\} \end{array}$$ (4)

where, *Cons* = 1000000 is a fixed constant such that decimal floating points can be converted into integers and *N* = 127 and $\overset{\left\lfloor \cdot \right\rfloor }{=}$ indicates to take the integer part of each $-\frac{Cons}{\log_{2}N}p_{i}\log_{2}p_{i}$. Noteworthily, for each cardiac cycle in screening, this pre-calculated *PiMap* permits the sole operation by picking the straightforward integer (i.e., *PiMap*[*i*]) from the set *PiMap* in accordance with the index *i* rather than calculating $-\frac{1}{\log_{2}N}p_{i}\log_{2}p_{i}$ using arithmetic and logarithmic operations. The use of *PiMap* remarkably decreases calculation times.

#### Algorithm implementation of H" (**A**)

Likewise, a buffer array *nu*_*wv*_*i*__ (0 ⩽ *wv*_*n*_ ⩽ 262143) is firstly defined to store the total number of the *i*-th characteristic element *wv*_*i*_ in space **A**. For a specific **A**, let *wv*_*n*_ be the element that will slide in **A** (i.e., *wv*_*n*_ will be the rightmost element in **A**), the present leftmost element in **A** is *wv*_*n*−127_ (i.e., *wv*_*n*−127_ will depart from **A** when *wv*_*n*_ slide in **A**). Because the variation of SE 𝓗′ (**A**) is entirely dependent upon the total numbers of slide-in element *wv*_*n*_ and slide-out element *wv*_*n*−127_ in the dynamic space **A** (they are *nu*_*wv*_*n*__ and *nu*_*wv*_*n*−127__, respectively), the outcome H' (**A**) can be calculated using a recursive method, see [19] for the definition of H' (**A**). In addition, the probabilities of *nu*_*wv*_*n*−127__ and *nu*_*wv*_*n*__ can be separately determined with a stepwise procedure rather than being processed concurrently. Therefore, a more concise method for calculating the H' (**A**) is proposed as follows,

$$\begin{array}{cc} & \;\;❶\;\;\text{if}\;nu_{wv_{n-127}}>0\\ & \;\;\;\;\;\;\;\;\;\{\;\;\;\;\;\;sh_{n}^{{}^{\prime }}\;-=PiMap\left[nu_{wv_{n-127}}\right];\\ & \;\;\;\;\;\;\;\;\;\;\;\;\;\;\;\;nu_{wv_{n-127}}--;\\ & \;\;\;\;\;\;\;\;\;\;\;\;\;\;\;\;sh_{n}^{{}^{\prime }}\;+=PiMap\left[nu_{wv_{n-127}}\right];\\ & \;\;\;\;\;\;\;\;\;\;\;\;\;\;\;\;\text{if}\;nu_{wv_{n-127}}\equiv 0\;\;\;\;\;k--;\;\;\;\;\;\;\;\}\;\; \end{array}$$

$$\begin{array}{cc} & \;\;❷\;\;\;\text{if}\;nu_{wv_{n}}\equiv 0\;\;\;\;\;k++;\\ & \;\;\;\;\;\;\;\;\;sh_{n}^{{}^{\prime }}\;-=PiMap\left[nu_{wv_{n}}\right];\\ & \;\;\;\;\;\;\;\;\;nu_{wv_{n}}++;\\ & \;\;\;\;\;\;\;\;\;sh_{n}^{{}^{\prime }}\;+=PiMap\left[nu_{wv_{n}}\right]; \end{array}$$

$$\begin{array}{cc} & \;\;❸\;\;\;\;sh_{n}^{{}^{\prime \prime }}=\frac{k}{127000000}sh_{n}^{{}^{\prime }}; \end{array}$$

where $sh_{n}^{{}^{\prime }}$ represents the H' (**A**) in Eq (7) in [19], and $sh_{n}^{{}^{\prime \prime }}$ represents the H" (**A**) herein; note that we fix *PiMap*[*i*] = 0 for the case *i* ≡ 0; and 127000000 = N*Cons = 127 × 1000000. As before, *k* is the total number of unique elements in **A**. In steps ❶–❸, *μ* − = *ν* and *μ* + = *ν* indicate *μ* = *μ* − *ν* and *μ* = *μ* + *ν*, respectively; *μ* − − and *μ* + + indicate *μ* = *μ* − 1 and *μ* = *μ* + 1, respectively (*μ* and *ν* indicate integer variables). The step ❶ is designed for dealing with the slide-out element *wv*_*n*−127_, and step ❷ is applied for dealing with the slide-in element *wv*_*n*_. The last step ❸ is used for calculating the $sh_{n}^{{}^{\prime \prime }}$. For the next slide-in word *wv*_*n*+1_, steps ❶–❸ are again executed to obtain the $sh_{n+1}^{{}^{\prime \prime }}$. From an online processing perspective, the inherent time delays of $sh_{n}^{{}^{\prime \prime }}$ are 63.5 and 65 points with respect to the *wv*_*n*_ and *hr*_*n*_, respectively.

**Fig 2:**
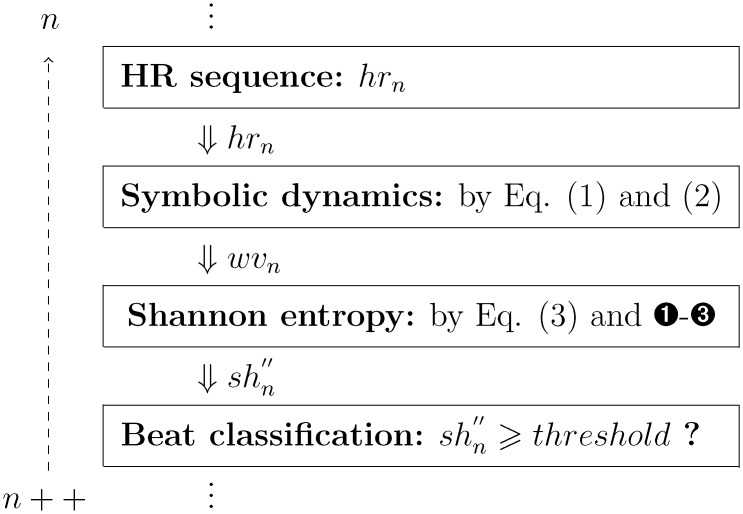
Overview of the beat-by-beat AF detection algorithm.

The parameters (*sy*_*n*_, *wv*_*n*_, *nu*_*wv*_*n*__, $sh_{n}^{{}^{\prime }}$ and *k*) are initially set to zero. A flowchart of the recursive realization of this method can be seen in Fig 2. Presumably, for each cardiac cycle screening, by using recursive algorithms, this AF detector consists of a very few basic operations, such as integer addition, subtraction, comparison and shifting operations. In fact, the calculation of $sh_{n}^{{}^{\prime \prime }}$ and distinguishing the current cardiac beat *hr*_*n*_, only require to include 1 multiplication and 1 division lying within $\frac{k}{127000000}\cdot$, together with 1 floating-point comparison between $sh_{n}^{{}^{\prime \prime }}$ and the decision-making threshold value. Therefore, a significant computational efficiency can be obtained.

This AF detector is investigated with four independently publicly-accessible sets of clinical ECGs (the Long-Term AF Database [LTAFDB], the MIT-BIH AF Database [AFDB], the MIT-BIH Arrhythmia Database [MITDB], and the MIT-BIH Normal Sinus Rhythm Database [NSRDB]) [23]. The LTAFDB database is designed as an initial training set, while the other three databases are used as testing sets. A brief summarization of these databases can be found in [19]. In order to achieve an unbiased assessment, all reference annotations of four databases are examined without any pre-manipulation. All tests are conducted with the use of C++ programming language.

## Results

### Performance metrics

This new AF detector and the existing methods are investigated in terms of sensitivity (*Se*), specificity (*Sp*), positive predictive value (*PPV*), and overall accuracy (*ACC*). For a specific database, we figure out the number of true positives (*TP*), true negatives (*TN*), false positives (*FP*), and false negatives (*FN*), and then the *Se*, *Sp*, *PPV*, and *ACC* are calculated by,

$$\begin{array}{cc} Se & =\frac{TP}{TP+FN},\;PPV=\frac{TP}{TP+FP},\\ Sp & =\frac{TN}{TN+FP},\;ACC=\frac{TP+TN}{TP+TN+FP+FN} \end{array}$$ (5)

We let *Se* and *Sp* as the mainstay quality parameters, while *PPV* and *ACC* are complementary.

### Results of the training database

**Fig 3:**
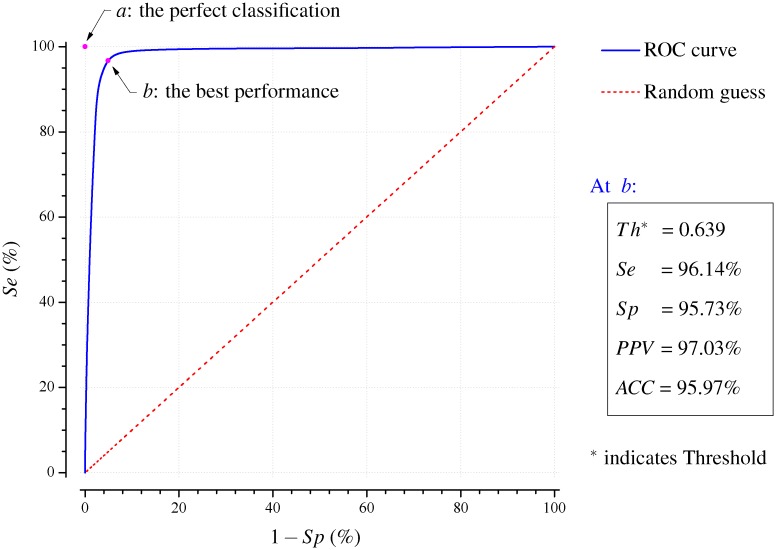
Receiver operating characteristic for the present algorithm when the training set of LTAFDB database is applied with threshold values from 0.0 to 1.0 in increments of 0.001. The calculated value for the area under the blue curve is 0.9845.

Prior to the performance investigation, a decision-making threshold value should be determined to best separate the AF and non-AF episodes. LTAFDB set is thus used for training the newly presented AF detector. This database consists of 84 long-term ECG recordings (typically 24 to 25 hours duration) of subjects with paroxysmal or sustained AF. It includes nearly 9 million cardiac beats of which 59.2% are annotated with AF. The discrimination threshold for H" (**A**) is tested from 0.0 to 1.0 in increments of 0.001 for the training set, and the parameters *Se*, *Sp*, 1 − *Sp*, *PPV* and *ACC* are then calculated for each threshold setting. The ROC curve is subsequently performed as shown in Fig 3, in which the *Se* is plotted in dependence of 1 − *Sp*. In the ROC space of Fig 3, *a* is the point of the ideal classification assuming that the automatic annotations of AF and non-AF are both 100% correct, and *b* is the point of the best performance of our method on the ROC curve, at which it has the shortest Euclidean distance to *a*. We can thus determine the parameters at position *b*, where the decision-making threshold is 0.639, and the distance is 0.0576 in ROC space and the area under ROC curve is 0.9845; the corresponding values of *Se*, *Sp*, *PPV* and *ACC* are 96.14%, 95.73%, 97.03% and 95.97%, respectively. When compared with our previous method based on the RRI [19], of which, the area under ROC curve is 0.9829 (+0.0016) and the Euclidean distance is 0.0594 (-0.00180) in the ROC space; the *Se*, *Sp*, *PPV* and *ACC* are 96.72% (-0.58%), 95.07% (+0.66%), 96.61% (+0.42) and 96.05% (-0.08%) for the best performing threshold of 0.353, respectively. Therefore, the present method demonstrates a slightly better performance.

### Results of the testing databases

The derived decision-making threshold (i.e., 0.639) is subsequently applied in this new AF detector when it is used across all testing databases: AFDB, MITDB and NSRDB sets. For our newly presented method, the statistical results from the testing databases are listed in Table 1.

**Table 1:** Summary of classification performance for three different testing databases with various cases (with the threshold of 0.639).

**Method**	**Feature**	**Year**	**Database**	**Key techniques**	**Results**			
					***Se*(%)**	***Sp*(%)**	***PPV*(%)**	***ACC*(%)**
This method	HR	2015	AFDB	Symbolic dynamics+Shannon Entropy	97.37	98.44	97.89	97.99
			AFDB†		97.31	98.28	97.89	97.84
			AFDB‡		98.43	98.46	97.92	98.45
			MITDB		97.83	87.41	47.67	88.51
			NSRDB		NA	99.68	NA	NA
			AFDB+NSRDB		97.36	99.32	96.86	98.98
			AFDB†+NSRDB		97.31	99.31	96.83	98.96
			AFDB‡+NSRDB		98.43	99.35	96.82	99.19

^†^ Records “00735” and “03665” excluded.

^‡^ Records “04936” and “05091” excluded.

‘NA’ indicates not applicable because there is no beat with AF reference annotation in this database.

**Fig 4:**
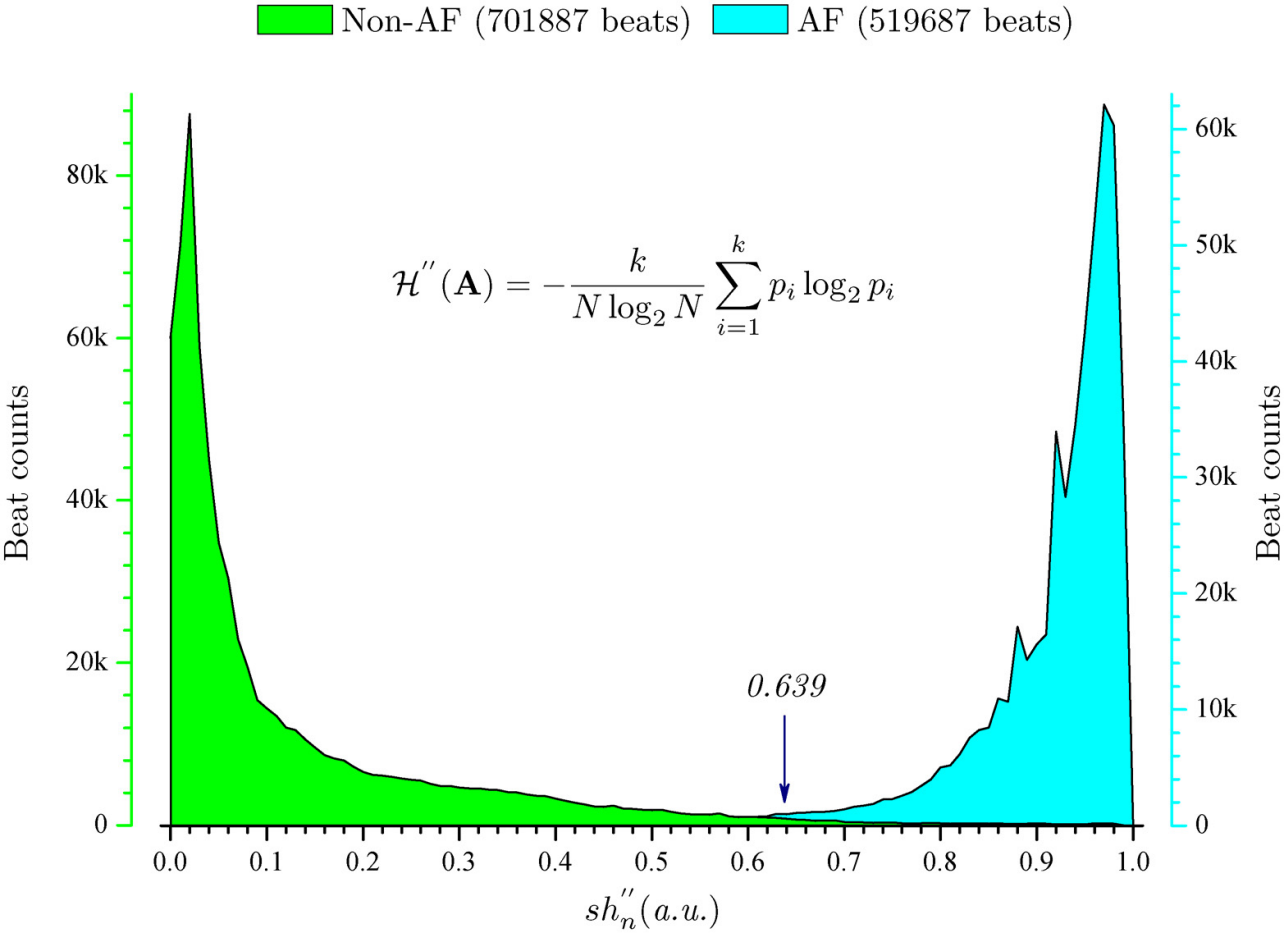
Probability histogram for annotated AF and non-AF beats of AFDB set.

Specifically, of the AFDB set, the calculated *Se*, *Sp*, *PPV* and *ACC* parameters are 97.37%, 98.44%, 97.89% and 97.99%, respectively. The values of SE H" (**A**) for AF (519687 beats) and non-AF (701887 beats) annotations in the AFDB set (a total of 1221574 beats for all 25 records) can be seen in Fig 4. It is clear that H" (**A**) discriminates AF well since the threshold value (i.e., 0.639) is very close to the cross point of the histogram of non-AF annotations and the histogram of AF annotations, see the arrow mark in Fig 4 for clarification. There is only a slight overlap between distributions of AF and non-AF annotations. Regarding the AFDB^†^ set (^†^ indicates records “00735” and “03665” excluded), the parameters are 97.31%, 98.28%, 97.89% and 97.84%, respectively. For the AFDB^‡^ set (^‡^ indicates records “04936” and “05091” excluded), the parameters are 98.43%, 98.46%, 97.92% and 98.45%, respectively. As others have shown [18], the records “04936” and “05091” include many incorrect manual AF annotations, these four parameters on AFDB^‡^ set are thus higher than those on AFDB and AFDB^‡^, respectively.

The MITDB set includes many coexisting various types of complex arrhythmias. The *Se* (97.83%) means most true AF beats are correctly detected. However, the *Sp* (87.41%) is a little low as several other arrhythmias are detected as AF. It thus incurs a few false alarms. Thereby, *PPV* (47.67%) and *ACC* (88.51%) are relatively low.

Records in the NSRDB set had no significant arrhythmias, the only calculated parameter *Sp* is 99.68% for this database since there is no AF annotation in this set. The *Sp* is high which implies most true negatives are correctly classified, and then produce a very high degree of predictive accuracy.

Regarding the combination cases of different databases, the *Se*, *Sp*, *PPV* and *ACC* values are 97.36%, 99.32%, 96.86% and 98.98%, respectively for AFDB+NSRDB set, and 97.31%, 99.31%, 96.83% and 98.96% for AFDB^†^+NSRDB set, and 98.43%, 99.35%, 96.82% and 99.19% for AFDB^‡^+NSRDB set, respectively. They perform almost as well as those on AFDB, AFDB^†^ and AFDB^‡^ sets. Because *Sp* of 99.68% (≈ 100%) is obtained for NSRDB set, the performances on these combination cases of the AFDB (AFDB^†^ and AFDB^‡^) and NSRDB sets are predominantly determined by detection rates on AFDB, AFDB^†^ and AFDB^‡^ sets, respectively.

## Discussion

Table 2 outlines a brief set of disclosed results of the state-of-the-art AF detection methods in literature. For more information on detection methods of AF, see elsewhere for detailed comparison of accuracy of developed methods prior to 2013 [19], [22], [24].

**Table 2:** Overview of performance comparison of previous algorithm using the same databases.

**Method**	**Features**	**Year**	**Database**	**Key techniques**	**Results**			
					*Se*(%)	*Sp*(%)	*PPV*(%)	*ACC*(%)
Petrėnas, *et al*[20]	RRI	2015	AFDB	Ectopic beat filtering+bigeminal suppression+signal fusion	97.12	98.28	–	–
			AFDB†		97.1	98.1	–	–
			AFDB‡		98.0	98.2	–	–
			MITDB		97.8	86.4	–	–
			NSRDB		NA	98.6	NA	NA
			AFDB+NSRDB		97.1	98.5	–	–
			AFDB†+NSRDB		96.8	98.2	–	–
			AFDB‡+NSRDB		97.3	98.2	–	–
Zhou, *et al*[19]	RRI	2014	AFDB	Nonlinear filter+integer filters+symbolic dynamics+Shannon Entropy	96.89	98.25	97.62	97.67
			AFDB†		96.82	98.06	97.61	97.50
			AFDB‡		97.83	98.19	97.56	98.04
			MITDB		97.33	90.78	55.29	91.46
			NSRDB		NA	98.28	NA	NA
			AFDB+NSRDB		96.89	98.27	92.30	98.03
			AFDB‡+NSRDB		97.53	98.26	90.09	98.16
Lee, *et al*[18]	RRI	2013	AFDB‡	TVCF+Shannon Entropy	98.22	97.68	–	97.91
			MITDB		91.1	89.7	–	–
			NSRDB		NA	99.7	NA	NA
See elsewhere for more disclosed results prior to 2013 [19], [22], [24]								

^†^ Records “00735” and “03665” excluded.

^‡^ Records “04936” and “05091” excluded.

‘–’ indicates without report. ‘NA’ indicates not applicable because there is no beat with AF reference annotation in this database. See text or relevant literature for abbreviation.

Petrėnas, *et al* [20] developed a promising AF detector with the utilization of ectopic beat filtering, bigeminal suppression and signal fusion techniques. The authors believed that a major advantage of their method was to be used in detecting short durations of AF episode. However, detailed information about the application of that method on the short durations of AF episode was not available. For the AFDB set, the *Se* and *Sp* values were 97.12% (+0.25%) and 98.28% (+0.16%), respectively. For the AFDB^†^ set, the *Se* and *Sp* values were 97.1% (+0.21%) and 98.1% (+0.18%), respectively. For the AFDB^‡^ set, the *Se* and *Sp* values were 98.0% (+0.43%) and 98.2% (+0.26%), respectively. For these three sets, our newly designed method shows a slightly better performance. For the MITDB set, the *Se* and *Sp* values were 97.8% (+0.03%) and 86.4% (+1.01%), respectively. We see that the *Se* of our method matches that of this method, but *Sp* of our method is distinctly better than that of this method. For the NSRDB set, the *Sp* was 98.6% (+1.08%) which indicates that our method has fewer number of false alarms. For the combination cases of AFDB (AFDB^†^ and AFDB^‡^) and NSRDB sets, our method also demonstrates certainly better performance, see Tables 1 and 2 for details.

We also compare the test results of this new AF detector with our early work in which the AF classifier employed RRI information along with the application of nonlinear/integer filters, symbolic dynamics as well as the Shannon entropy [19]. For the AFDB set, the *Se*, *Sp*, *PPV* and *ACC* parameters were 96.89% (+0.48%), 98.25% (+0.19%), 97.62% (+0.27%) and 97.67% (+0.32%), respectively. For AFDB^†^ set, these parameters were 96.82% (+0.49%), 98.06% (+0.22%), 97.61% (+0.28%) and 97.50% (+0.34%), respectively. For AFDB^‡^ set, these parameters were 97.83% (+0.60%), 98.19% (+0.27%), 97.56% (+0.36%) and 98.04% (+0.41%), respectively. This new method shows a better performance on AFDB set cases. It is worth noting that for the MITDB set, these parameters were 97.33% (+0.50%), 90.78% (−3.37%), 55.29% (−7.62%) and 91.46% (−2.95%), respectively. Although this new method has a higher *Se*, it has significantly poorer *Sp*, which is possibly because different strategies were used in symbol definition of symbolic dynamics for the two methods. For the NSRDB set, the *Sp* was 98.28% (+1.40%) which indicates this new method has a better performance. The results in Tables 1 and 2 indicate that for the combination cases of AFDB (AFDB^†^ and AFDB^‡^) and NSRDB sets, this new method also demonstrates better performance since performances on these combination cases are vastly determined by the detection capability on the AFDB set cases. The preliminary results are promising but improvements are still necessary before finalizing the algorithm.

Lee, *et al* introduced a RRI based method to identify AF [18], which was based upon the time-varying coherence functions (TVCF) and the Shannon entropy (SE). By choosing the AFDB^‡^ set for evaluation, the calculated *Se*, *Sp* and *ACC* values were 98.22% (+0.21%), 97.68% (+0.78%) and 97.91% (+0.54%), respectively. We see that our new method outperforms this method on the AFDB^‡^ set. Using the MITDB set, the *Se* and *Sp* values were 91.1% (+6.73%) and 89.7% (−2.29%), respectively. Our method has a markedly higher *Se*, whereas a lower *Sp*. From a standpoint of the early prevention and intervention of possible ischemic stroke, the *Se* is more important than the *Sp*. With regard to the NSRDB set, the *Sp* was 99.7% (−0.02%), which indicates these two methods have consistent performances for this database. From the perspective of computational complexity, the TVCF and SE based AF detector involved many convolution operations and the Fourier transformation with floating-point operations, consequently posing a computational challenge of the timing of successive detections.

**Fig 5:**
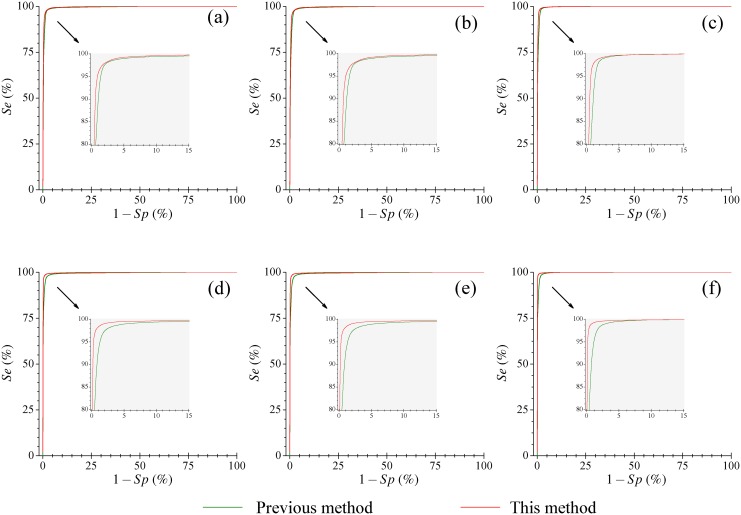
Receiver operating characteristic (ROC) curves for the present and our previous algorithms when the AFDB, AFDB^†^, AFDB^‡^ and NSRDB databases are tested with various situations. (A) ROC curves of the AFDB set; (B) ROC curves of the AFDB^†^ database (^†^ indicates records “00735” and “03665” excluded); (C) ROC curves of the AFDB^‡^ database (^‡^ indicates records “04936” and “05091” excluded); (D) Results of the AFDB+NSRDB database, (E) Results of the AFDB^†^+NSRDB database and (F) Results of the AFDB^‡^+NSRDB database. The calculated values for the area under the curves are listed in Table 3.

By utilization of the same decision-making threshold (of 0.639), the performance of the present AF detector is investigated on three clinical databases under different situations. This might misleadingly imply that the metrics *Se*, *Sp*, *PPV* and *ACC* are completely dependent on the threshold for each databases, and the differences in performance from previous methods might only be a result of statistical scatter. However, the “statistical scatter” is a random event in Statistics, the performances of this newly presented method are independently tested on different clinical databases with various situations (total 33 parameters are obtained). It is worth noting that almost all of these 33 parameters are better than that of pervious methods which definitely indicate that the performance of this new method is better than previous methods. Another spontaneous question may arise regarding how about the performance when using different threshold settings in this AF detector for various databases. It is well know that the area under the receiver operating characteristic (ROC) curve is a better threshold independent metrics for performance evaluation at various thresholds. However, as far as the AF detection concerned, it is very difficult to apply this metric for performance assessment of different algorithms, because some methods were implemented in time domain, some methods were implemented in frequency domain, and some were implemented in the domains defined by the authors themselves. In addition, some methods were implemented without the standard training process but with a empirical threshold. Nevertheless, we can compare this new method with our previously proposed method [19], since these two methods are based on the coarser version of Shannon entropy. We then quantify the performances of two method with various decision-making threshold settings for each testing database. Discrimination threshold values from 0.0 to 1.0 in increments of 0.001 are assessed on the AFDB, AFDB^†^, AFDB^‡^ and NSRDB databases with various situations; we can thus obtain the ROC curves and calculate the areas under the ROC curves. The corresponding test ROC curves of two methods are displayed in Fig 5, in which, each inset represents results of a database or a combined databases; 2 curves in each inset stand for ROC curves of previous and this methods, respectively. Table 3 lists the areas of two ROC curves shown in each inset of Fig 3. We clearly see that the performance of this new method is better than that of our previous method. Furthermore, findings from these ROC curves reciprocally offer additional insights to help select a decision-making threshold value for the AF determination.

**Table 3:** The areas under receiver operating characteristic (ROC) curves for the present and our previous algorithms when AFDB, AFDB^†^, AFDB^‡^ and NSRDB databases are tested with various situations.

Databases	Areas under the ROC curves	
	This method	In [19]
AFDB	0.9965	0.9944
AFDB†	0.9962	0.9940
AFDB‡	0.9975	0.9958
AFDB+NSRDB	0.9980	0.9949
AFDB†+NSRDB	0.9980	0.9948
AFDB‡+NSRDB	0.9989	0.9970

^†^ Records “00735” and “03665” excluded.

^‡^ Records “04936” and “05091” excluded.

### An investigation on computational complexity

This new AF detector is implemented using the C++ programming language. For a single cardiac beat in screening, the computational complexity of our method is analyzed: (*i*) for the best case, it only has one integer comparison and one integer assignment in Eq (1), and two integer subtractions, two integer additions and two integer-shifting operations in Eq (2), and two integer comparisons, one integer subtraction, one integer ‘ − =’ operation, one integer ‘+=’ operation, two integer ‘++’ operations, one integer multiplication, one floating-point division operation and one floating-point assignment in ❶–❸; (*ii*) for the worst case, it only has one integer comparison, one integer division, one integer-round operation and one integer-assignment operation in Eq (1), and two integer subtractions, two integer additions, two integer-shifting operations and one integer assignment in Eq (2), and three integer comparisons, one integer subtraction, two integer ‘ − =’ operations, two integer ‘+=’ operations, two integer ‘−−’ operations, two integer ‘++’ operations, one integer multiplication, one floating-point division operation and one floating-point assignment in ❶–❸. Therefore, a very low computational complexity can be achieved. Table 4 displays the computation time taken by this method while comparing with the method we developed in [19]. It is apparent that computation time of this method is diminished approximately 40 percent of that in [19] for each database. In our previous work, we have attested the RRI based online AF detection method had a high level of computational efficiency. Nevertheless, computational efficiency of this HR based method is significantly better than that of the RRI based method [19]. This implies that the newly presented method is quite suitable in real-time, long-term monitoring. In addition, this method provides certain benefit in remote cloud computing.

**Table 4:** The computation time of the processing of this method.

**Databases**	**Signal duration (sec)**	**Computation time (sec)§**	
		**This method**	In [19]
LTAFDB	6970560 (1936.27 hours)	6.434	11.09
AFDB	917052.96 (254.74 hours)	0.872	1.445
AFDB†	843688.72 (234.36 hours)	0.774	1.353
AFDB‡	843688.72 (234.36 hours)	0.811	1.406
MITDB	86666.67 (24.07 hours)	0.0834	0.116
NSRDB	1574976 (437.49 hours)	1.139	1.825
AFDB+NSRDB	2492028.96 (692.23 hours)	1.757	3.258

^†^ Records “00735” and “03665” excluded.

^‡^ Records “04936” and “05091” excluded.

^§^ Desktop test environment: (a) hardware: Intel Pentium(R) Dual-Core E5800(3.20GHz)/DDR3 RAM (2GBytes, 800MHz)/ HDD(7200rpm); (b) software: WINDOWS XP Professional(in [19])/WINDOWS 7 Professional(in this method)/mingw32-g++/C++. The computation times are the average values of 100 trials, and they include the time consumption for importing annotation data from the HDD into the RAM.

The accuracy of estimation of the probability of word *wv*_*n*_ distribution is critically dependent on the adequacy of width of the analysis bin (i.e., *N* herein). A small amount of words inside a small bin (≪ *N*) might incur a poor estimation [25], and then can cause false alarms; the size of bin *N* for calculation of H" (**A**) was thus empirically set to 127 in the present study. However, we have observed that most of the AF episodes of relatively short duration (e.g., around decades of seconds) in these four databases were correctly classified. Nevertheless, for sporadic AF episodes of very short duration (e.g., less than 20 seconds), it might incur false negative detection. From this respect, this may be a possible limitation. As stated previously, we also reiterate the fact that a small *Sp* calculated from the MITDB set implies that this new AF detection method could be further refined when various complicated arrhythmias are coexistent; in the meanwhile, we hope that this study will stimulate further discussion and innovation such that a more robust AF detector can be developed to deal with these clinical cases.

In summary, the present AF detector takes advantage of straightforwardly defined symbols in the original HR sequence, and a very low computational complexity is obtained. The combination of symbolic dynamics and SE produces a more robust and more concise AF capture method. This could be incorporated into ECG interpretation computerized systems to improve the reliable classification of supraventricular tachyarrhythmia regarding the use of noninvasive cardiac rhythm monitoring.

## Conclusions

The higher sensitive and more accurate detection algorithms are clinically desirable for the attainment of quick differentiation of AF episodes in ECG recordings. This study is a pilot trial that employs the HR as a key feature for the accurate AF discrimination. It is also worth mentioning that the present detection method doesn’t require any digital filters (i.e., convolution), and a real-time, low-complexity screening can be achieved by operating with a pre-determined set of the probability of word *wv*_*n*_ distribution (i.e., $-\frac{1}{\log_{2}N}p_{i}\log_{2}p_{i}$) as well as clever recursive realization of the symbolic dynamics and SE. In effect, a very few arithmetical operations are required for AF identification per heartbeat. Our new method is investigated on four publicly-accessible sets of clinical data with different situations. The well established and understood statistical parameters *Se*, *Sp*, *PPV* and *ACC* are calculated for each data set, and compared with those of the latest AF detection methods. Taken together, the newly presented method outperforms the traditional methods. The AF is firmly established as a risk factor for ischemic-type stroke, and there is a high incidence of morbidity caused by the acute ischemic stroke in non-hospitalized patients. A principal concern in health informatics is to research and develop techniques that enable the reliable identification of AF at an early stage. We believe that this method would be integrated into wearable devices, such as ECG mini-machine, smart phone and smart watch, for the AF episode screening in outside of clinical settings in the near future.
